# Development of a new software tool and analysis method to improve determination of G6PD status

**DOI:** 10.1186/1475-2875-13-S1-P48

**Published:** 2014-09-22

**Authors:** Michael Kalnoky, Maria Kahn, Sampa Pal, Nicole LaRue, Brandon Leader, Germana Bancone, Francois Nosten, Gonzalo J Domingo

**Affiliations:** 1 Tsuga Analytics, Seattle, WA, USA; 2 PATH, Diagnostic Technologies, Seattle, WA, USA; 3 Shoklo Malaria Research Unit, Mae Sot, Thailand

## 

8-aminoquinoline drugs are critical to malaria elimination campaigns due to their unique ability to kill the dormant liver stage form of *Plasmodium vivax*. Due to the potential for hemolytic reactions in G6PD-deficient patients, safe administration of these drugs requires assessing a person’s glucose-6-phosphate dehydrogenase (G6PD) status. Quantitative and qualitative phenotypic tests that assess overall G6PD activity within a blood sample are generally able to distinguish between G6PD-normal and deficient patients, not factoring in red blood cell (RBC) count and hemoglobin level. A cytofluorimetric assay (Shah *et al.* 2012) allows for screening of G6PD activity within individual RBCs and identifies heterozygous females. This method enables the discrimination of distinct cell populations based on their G6PD activity, estimating the actual proportion of deficient RBCs that are likely to hemolyze during drug treatment. As part of the study, we enhanced the G6PD classification method by analyzing data from the cytofluorimetric assay with a software tool developed at PATH. This software tool not only mitigates for variations in measurements generated by different flow cytometers, but also implements a metric for a more quantitative interpretation of cytochemical staining results. PATH developed the software tool by correlating enzyme activity (G-6-PDH Kit, Trinity Biotech), genotypic information, and complete blood cell count data with the distinct G6PD activity measured by flow cytometry. As an additional level of validation, the software tool is undergoing blind testing with several hundred subsequent samples collected from multiple international sites. PATH plans to make the software publicly available and hosted online to aid clinical and research studies in determining G6PD status.

